# National trends in utilization and outcomes of coronary revascularization procedures among people with and without type 2 diabetes in Spain (2001–2011)

**DOI:** 10.1186/1475-2840-13-3

**Published:** 2014-01-03

**Authors:** Ana Lopez-de-Andres, Rodrigo Jimenez-García, Valentin Hernandez-Barrera, Napoleon Perez-Farinos, Jose M de Miguel-Yanes, Manuel Mendez-Bailon, Isabel Jimenez-Trujillo, Angel Gil de Miguel, Carmen Gallardo Pino, Pilar Carrasco-Garrido

**Affiliations:** 1 Preventive Medicine and Public Health Department, Health Sciences Faculty, Rey Juan Carlos University, Madrid, Spain; 2 Agencia Española de Seguridad Alimentaria y Nutrición, Ministerio de Sanidad, Servicios Sociales e Igualdad, Madrid, Spain; 3 Servicio de Medicina Interna, Hospital Universitario del Sureste, Madrid, Spain; 4 Servicio de Medicina Interna, Hospital Clínico San Carlos, Madrid, Spain

**Keywords:** Type 2 diabetes, Percutaneous coronary intervention, Coronary artery bypass graft surgery, Hospitalization, Length of stay, In-hospital mortality

## Abstract

**Background:**

Diabetes is associated with a high risk of death due to coronary artery disease (CAD). People with diabetes suffering from CAD are frequently treated with revascularization procedures. We aim to compare trends in the use and outcomes of coronary revascularization procedures in diabetic and non-diabetic patients in Spain between 2001 and 2011.

**Methods:**

We identified all patients who had undergone coronary revascularization procedures, percutaneous coronary interventions (PCI) and coronary artery bypass graft (CABG) surgeries, using national hospital discharge data. Discharges were grouped by diabetes status: type 2 diabetes and no diabetes. The incidence of discharges attributed to coronary revascularization procedures were calculated stratified by diabetes status. We calculated length of stay and in-hospital mortality (IHM). We apply joinpoint log-linear regression to identify the years in which changes in tendency occurred in the use of PCI and CABG in diabetic and non-diabetic patients. Multivariate analysis was adjusted by age, sex, year and comorbidity (Charlson comorbidity index).

**Results:**

From 2001 to 2011, 434,108 PCIs and 79,986 CABGs were performed. According to the results of the joinpoint analysis, we found that sex and age-adjusted use of PCI increased by 31.4% per year from 2001 to 2003, by 15.9% per year from 2003 to 2006 and by 3.8% per year from 2006 to 2011 in patients with diabetes. IHM among patients with diabetes who underwent a PCI did not change significantly over the entire study period (OR 0.99; 95% CI 0.97-1.00).

Among patients with diabetes who underwent a CABG, the sex and age-adjusted CABG incidence rate increased by 10.4% per year from 2001 to 2003, and then decreased by 1.1% through 2011. Diabetic patients who underwent a CABG had a 0.67 (95% CI 0.63-0.71) times lower probability of dying during hospitalization than those without diabetes.

**Conclusions:**

The annual percent change in PCI procedures increased in diabetic and non-diabetic patients. Higher comorbidity and the female gender are associated with a higher IHM in PCI procedures. In diabetic and non-diabetic patients, we found a decrease in the use of CABG procedures. IHM was higher in patients without diabetes than in those with diabetes.

## Background

Diabetes is associated with a high risk of death due to coronary artery disease (CAD). Current figures indicate that cardiovascular events are responsible for 80% of all deaths in patients with diabetes [1]. In Spain, 75% of patients with diabetes die primarily of CAD [2]*.* Recent studies reported declines in cardiovascular mortality in patients with diabetes [3-6], which has been attributed to better management of risk factors; however, these have been less pronounced than in those without diabetes [7].

People with diabetes represent an increasing proportion of CAD patients, many of whom are treated with revascularization procedures [8]. Approximately 25% of all coronary revascularization procedures performed each year in the US are done on patients with diabetes [9]. Coronary revascularization for patients with diabetes can be achieved using coronary artery bypass graft (CABG) surgeries or percutaneous coronary interventions (PCIs) [10]. While PCI is more commonly used in patients affected by single-vessel CAD, the best strategy for patients with advanced CAD is still debated, due to a higher repeat revascularization rate at 1-year follow-up in patients treated using PCI [11].

Kappetein AP et al. reported the 5-year results of the SYNTAX trial with regard to patients with diabetes. Of 1800 patients in the SYNTAX trial, 452 had diabetes. In this group of diabetic patients, 5-year rates for major adverse cardiac and cerebrovascular events (46 vs. 29%; P < 0.001) and repeat revascularization (35 vs. 15%; P < 0.001) were significantly higher for PCI vs. CABG [12].

The FREEDOM Trial Investigators found that, in patients with diabetes and advanced CAD, CABG was superior to PCI using first generation drug-eluting stents. The benefit of CABG stemmed from the differences in the rates of both AMI (p < 0.001) and death from any cause (p = 0.049) [13].

Secular trends in the use of coronary revascularization procedures have been examined.

In the UK, Vamos et al. found that PCI rates increased significantly (IRR 1.01 [95% CI 1.005-1.03]) in people with diabetes from 2004–2009, whereas CABG rates declined [14]. However, there are no studies investigating national trends in the use of coronary revascularization procedures in people with diabetes in Spain.

In this study, we used national hospital discharge data to describe and compare trends in the use of coronary revascularization procedures in diabetic and non-diabetic patients between 2001 and 2011 in Spain. In particular, we analyzed trends in the use of CABG and PCI, patient comorbidities, and in-hospital outcomes such as length of stay and in-hospital mortality.

## Methods

A retrospective, descriptive, epidemiological study was conducted using the Spanish National Hospital Database (CMBD*, Conjunto Minimo Básico de Datos*), which compiles all public and private hospital data, hence covering more than 95% of hospital discharges [15]. The CMBD database is managed by the Spanish Ministry of Health, Social Services and Equality and includes patient variables (sex, date of birth), date of admittance, date of discharge, up to 14 discharge diagnoses, and up to 20 procedures performed during hospitalization. The Spanish Ministry of Health, Social Services and Equality sets recording standards and performs periodic audits [15].

We selected all surgical admissions of patients who underwent coronary revascularization procedures using the International Classification of Diseases - Ninth Revision, Clinical Modification (ICD-9-CM). The procedure codes used were: 36.10-36.19 for CABG and 36.06; 36.07; 36.09; 00.66 for PCI.

Discharges were grouped by diabetes status as follows: no diabetes and type 2 diabetes (ICD-9-CM codes: 250.x0; 250.x2). Patients with type 1 diabetes were excluded (ICD-9-MC codes: 250.x1; 250.x3).

Patients who underwent both CABG and PCI during their hospitalization were excluded.

The outcomes of interest included the percentage of patients who died during hospitalization, defined as in-hospital mortality (IHM) and the mean length of hospital stay (LOS).

Clinical characteristics included information on overall comorbidity at the time of surgery, which was assessed by computing the Charlson comorbidity index (CCI). The index applies to 17 disease categories whose scores are totaled to obtain an overall score for each patient [16]. The index is subsequently categorized into three levels: 0, no disease; 1, one or two diseases; and 2, three or more diseases [17]. To calculate the CCI, we used 17 disease categories, excluding diabetes and AMI, as described by Thomsen RW et al. [17].

### Statistical analysis

A descriptive statistical analysis was performed. Rates for type 2 diabetic and non-diabetic patients for each coronary revascularization procedure were calculated in terms of 100,000 inhabitants. We also calculated the yearly age- and sex-specific incidence rates for diabetic and non-diabetic patients, dividing the number of cases per year, sex, and age group by the corresponding number of people in that population group, according to data from the Spanish National Institute of Statistics, as reported on December 31 of each year [18].

In our study, we used joinpoint log-linear regression to identify the years in which changes in tendency occurred in the use of PCI and CABG in patients with and without type 2 diabetes, as well as to estimate the annual percentage of change (APC) in each of the periods delimited by the points of change. The analysis started with the minimum number of joinpoints and tested whether the inclusion of one or more joinpoints was statistically significant [19]. In the final model, each joinpoint indicated a significant change in the tendency, and the APC was obtained in each of the segments delimited by the joinpoints, using the weighted least squares technique. The Joinpoint Regression Program Version 4.0.4 was used for the analysis [20].

In order to test the time trend for IHM, logistic regression analyses were performed with mortality as a binary outcome, using year of discharge, sex, age, and CCI as independent variables. Models were generated for diabetic and non-diabetic subjects and for the entire population, in order to compare the IHM of those who have the disease and those who do not. Statistical analyses were performed using Stata version 10.1 (Stata, College Station, Texas, USA). Statistical significance was set at p < 0.05 (2-tailed).

### Ethical aspects

Data confidentiality was maintained at all times according to Spanish legislation. Patient identifiers were deleted before the database was provided to the authors, in order to maintain patient anonymity. It is not possible to identify patients at individual levels, either in this article or in the database. Given the anonymous and mandatory nature of the dataset, it was not necessary to obtain informed consent. The study protocol was approved by the ethics committee of the Universidad Rey Juan Carlos.

## Results

From 2001 to 2011, a total of 514,094 admissions of patients who underwent scheduled or unscheduled coronary revascularization procedures were recorded in Spain. Over the study period, 29.8% (n = 153,242) of all patients who underwent coronary revascularization procedures had type 2 diabetes. There were 434,108 PCIs (29.2% [n = 126,776] in patients with type 2 diabetes) and 79,986 CABGs (33.0% [n = 26,466] in patients with type 2 diabetes).

### Percutaneous coronary intervention

In patients who underwent a PCI, there was a significant male predominance in patients both with and without diabetes (69.5% and 79.9%). Mean age was 67.5 years (SD, 10.1 years) in patients with type 2 diabetes and 63.9 years (SD, 12.1 years) in those without diabetes (p < 0.01).

Patients with type 2 diabetes who underwent PCIs had higher CCI values compared to those without diabetes (37.2% vs. 27.1% with one or more coexisting conditions, respectively).

Among those who received a PCI, the median LOS was significantly higher in patients with type 2 diabetes (6.0 days [IQR 11.0 days]) compared to those without diabetes (5.0 days [IQR 7.0 days]). Also, IHM was significantly higher in patients with type 2 diabetes (2.5%) compared to patients without diabetes (2.0%).

**Figure 1:**
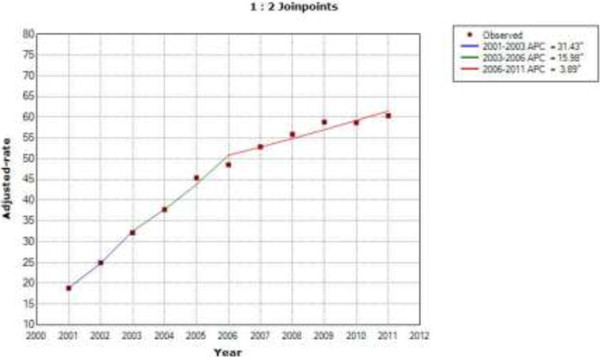
**Joinpoint analysis in annual PCI in patients with type 2 diabetes in Spain, 2001–2011.** Footnote: APC: Annual percent change (based on rates that were sex and aged-adjusted using the Spanish National Statistics Institute Census projections) calculated by using joinpoint regression analysis. ˆAPC is significantly different from zero (two-side P < 0.05).

**Figure 2:**
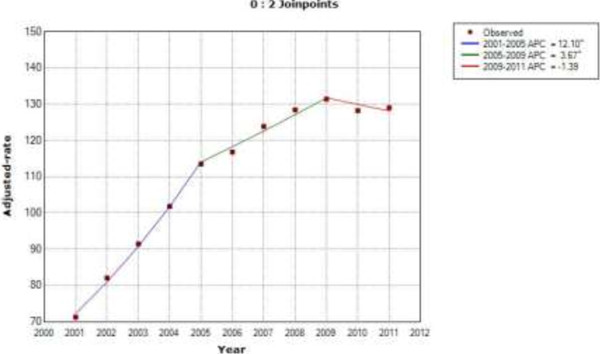
**Joinpoint analysis in annual PCI in patients without type 2 diabetes in Spain, 2001–2011.** Footnote: APC: Annual percent change (based on rates that were sex and aged-adjusted using the Spanish National Statistics Institute Census projections) calculated by using joinpoint regression analysis. ˆAPC is significantly different from zero (two-side P < 0.05).

According to the results of the joinpoint analysis, we found that sex and age-adjusted use of PCI increased by 31.4% per year from 2001 to 2003, by 15.9% per year from 2003 to 2006 and by 3.8% per year from 2006 to 2011 in patients with type 2 diabetes (Figure 1). In patients without diabetes, the use of PCI increased by 12.1% per year from 2001 to 2005 and by 3.6% per year from 2005 to 2009. From 2009 to 2011, it decreased by 1.39% per year, but not significantly (Figure 2).

Table 1 shows time trend outcomes in annual PCIs in patients with and without type 2 diabetes in Spain, 2001–2011. We found that the mean age of patients with diabetes who underwent a PCI was 66.1 ± 9.7 years in 2001 and increased to 68.2 ± 10.4 years in 2011 (P < 0.05), the proportion of men varied significantly from 67.1% in 2001 to 71.3% in 2011 and the prevalence of those with a CCI of one or more increased from 30.5% in 2001 to 41.1 (P < 0.05).

**Table 1:** Characteristics and outcomes of hospital discharges after percutaneous coronary intervention among patients with and without type 2 diabetes in Spain, 2001–2011

		**2001**	**2002**	**2003**	**2004**	**2005**	**2006**	**2007**	**2008**	**2009**	**2010**	**2011**
No diabetes	N	16200	18842	21438	24375	27745	29184	31607	33452	34840	34463	35186
	Incidence*	71.3	82.1	91.4	101.8	113.5	116.8	123.8	128.4	131.4	128.2	128.9
	Age§, mean	62.9	63.0	62.8	63.3	63.7	63.9	64.1	64.3	64.4	64.3	64.5
	(SD)	(11.4)	(11.6)	(11.7)	(11.7)	(11.8)	(11.9)	(12.0)	(12.2)	(12.3)	(12.4)	(12.5)
	Female ǂ, n	3039	3526	3971	4771	5542	5866	6353	6820	7199	7097	7389
	(%)	(18.7)	(18.7)	(18.5)	(19.5)	(19.9)	(20.1)	(20.1)	(20.3)	(20.6)	(20.5)	(21)
	CCI 0ǂ, n	12541	14250	16116	18102	20564	21687	22987	24070	24771	24362	24683
	(%)	(77.4)	(75.6)	(75.1)	(74.2)	(74.1)	(74.3)	(72.7)	(71.9)	(71.1)	(70.6)	(70.1)
	CCI 1-2ǂ, n	3561	4473	5152	6057	6928	7204	8265	8937	9574	9538	9948
	(%)	(21.9)	(23.7)	(24.0)	(24.8)	(24.9)	(24.6)	(26.1)	(26.7)	(27.4)	(27.6)	(28.2)
	CCI ≥ 3ǂ, n	98	119	170	216	253	293	355	445	495	563	55
	(%)	(0.6)	(0.6)	(0.7)	(0.8)	(0.9)	(1.0)	(1.1)	(1.3)	(1.4)	(1.6)	(1.5)
	LOS§, median	6.0	6.0	6.0	6.0	6.0	6.0	5.0	5.0	5.0	5.0	5.0
	(IQR)	(9.0)	(8.0)	(8.0)	(8.0)	(7.0)	(7.0)	(7.0)	(7.0)	(6.0)	(6.0)	(6.0)
	IHM ǂ, n	317	310	400	458	522	510	591	691	782	746	828
	(%)	(1.9)	(1.6)	(1.8)	(1.8)	(1.8)	(1.7)	(1.8)	(2.0)	(2.2)	(2.1)	(2.3)
Type 2 diabetes	N	4369	5847	7675	9188	11235	12276	13607	14662	15652	15807	16458
	Incidence*	18.8	24.9	32.1	37.7	45.3	48.5	52.8	55.8	58.8	58.6	60.3
	Age§, mean	66.1	66.5	66.5	66.8	67.0	67.4	67.7	67.9	67.8	68.2	68.2
	(SD)	(9.7)	(9.7)	(9.7)	(9.8)	(9.9)	(9.9)	(10.1)	(10.2)	(10.3)	(10.4)	(10.4)
	Female ǂ, n	1440	1937	2463	2852	3454	3853	4255	4577	4618	4579	4723
	(%)	(32.9)	(33.1)	(32.0)	(31.0)	(30.7)	(31.3)	(31.2)	(31.2)	(29.5)	(28.9)	(28.7)
	CCI 0ǂ, n	3034	3962	5040	5971	7266	8152	8586	8871	9405	9443	9691
	(%)	(69.4)	(67.7)	(65.6)	(64.9)	(64.6)	(66.4)	(63.1)	(60.5)	(60.0)	(59.7)	(58.8)
	CCI 1-2ǂ, n	1284	1805	2514	3067	3755	3916	4745	5428	5846	5915	6308
	(%)	(29.3)	(30.8)	(32.7)	(33.3)	(33.4)	(31.9)	(34.8)	(37.0)	(37.3)	(37.4)	(38.3)
	CCI ≥ 3ǂ, n	51	80	121	150	214	208	276	363	401	449	459
	(%)	(1.1)	(1.3)	(1.5)	(1.6)	(1.9)	(1.6)	(2.0)	(2.4)	(2.5)	(2.8)	(2.7)
	LOS§, median	7.0	7.0	7.0	7.0	7.0	6.0	6.0	6.0	6.0	6.0	6.0
	(IQR)	(10.0)	(10.0)	(9.0)	(9.0)	(8.0)	(8.0)	(7.0)	(7.0)	(7.0)	(7.0)	(6.0)
	IHM ǂ, n	85	157	186	201	289	314	332	403	405	419	425
	(%)	(1.9)	(2.6)	(2.4)	(2.1)	(2.5)	(2.5)	(2.4)	(2.7)	(2.5)	(2.6)	(2.5)

N: Number of procedures; LOS: length of stay; IHM: In-hospital mortality;

CCI (Charlson Comorbidity Index): Comorbidities included in the Charlson comorbidity index, except diabetes and AMI.

Incidence per 100.000. Incidence was calculated using the Spanish National Statistics Institute census projections adjusted by sex and age (16).

*P < 0.05 (Poisson regression analysis).

§P < 0.05 (ANOVA or Kruskal-Wallis analysis).

ǂP < 0.05(χ2 linear tend analysis).

LOS after PCI decreased significantly over the study period in both groups of patients (P < 0.01), showing higher values among those with type 2 diabetes in all years analyzed (Table 1).

The IHM among those who underwent a PCI increased for those without diabetes (1.9% in 2001 vs. 2.3% in 2011; P < 0.05) but remained stable for those suffering from type 2 diabetes (1.9% vs. 2.5%, P = 0.10) (Table 1).

As can been seen in Table 2, after multivariate adjustment, the IHM among patients with diabetes who underwent a PCI did not change significantly from 2001 to 2011 (OR 0.99; 95% CI 0.97-1.00). IHM was significantly higher in women than in men (OR 1.36; 95% CI 1.26-1.46) and was higher in those with one or two (OR 2.92; 95% CI 2.71-3.15) or three or more (OR 5.27; 95% CI 4.51-6.15) comorbidities associated with type 2 diabetes.

**Table 2:** Multivariate analysis of the factors associated with in-hospital mortality after coronary revascularization procedures among patients with and without type 2 diabetes in Spain, 2001-2011

		**In-hospital mortality (OR)**^**†**^		**In-hospital mortality (OR)**^**†**^		**In-hospital mortality (OR)**^**†**^	
		**Type 2 diabetes**		**No diabetes**		**All**	
		**PCI**	**CABG**	**PCI**	**CABG**	**PCI**	**CABG**
Age (years)	35-59	1	1	1	1	1	1
	60-69	1.49	1.65	1.45	1.47	1.46	1.50
		(1.30-1.70)	(1.35-2.01)	(1.33-1.58)	(1.31-1.63)	(1.36-1.57)	(1.37-1.65)
	70-79	2.38	2.69	2.30	2.33	2.34	2.40
		(2.10-2.71)	(2.23-3.25)	(2.14-2.48)	(2.10-2.57)	(2.19-2.49)	(2.20-2.63)
	≥80	3.73	3.55	3.93	3.42	3.88	3.44
		(3.25-4.29)	(2.72-4.62)	(3.62-4.28)	(2.99-3.91)	(3.61-4.16)	(3.05-3.88)
Sex	Men	1	1	1	1	1	1
	Female	1.36	1.69	1.34	1.53	1.35	1.58
		(1.26-1.46)	(1.52-1.89)	(1.26-1.42)	(1.42-1.65)	(1.29-1.41	(1.49-1.68)
Charlson index	0	1	1	1	1	1	1
	1-2	2.92	2.58	3.14	2.56	3.07	2.56
		(2.71-3.15)	(2.31-2.88)	(2.98-3.31)	(2.40-2.74)	(2.94-3.21)	(2.42-2.72)
	≥3	5.27	4.95	6.62	5.43	6.07	5.27
		(4.51-6.15)	(3.83-6.42)	(5.86-7.48)	(4.62-6.38)	(5.51-6.68)	(4.60-6.05)
Diabetes	No	-	-	-	-	1	1
	Yes	-	-	-	-	0.99	0.67
		-	-	-	-	(0.95-1.04)	(0.63-0.71)
Year		0.99	0.93	1.00	0.95	0.99	0.94
		(0.97-1.00)	(0.91-0.94)	(0.99-1.01)	(0.93-0.96)	(0.98-1.01)	(0.93-0.95)

^†^Calculate using logistic regression models: Odds Ratio (OR). The logistic regression multivariate model were built using as dependent variable”death (yes/no)” of PCI or CABG respectively, and as independent variables year, sex, Charlson comorbidity index and age.

### Coronary artery bypass graft

We found that, in patients who underwent a CABG, the mean age was significantly higher in patients with type 2 diabetes than in those without diabetes (67.3 years [SD 8.7 years] vs. 66.4 years [SD 10.1 years]) and there was higher proportion of males undergoing CABG procedures in both groups (73.3% in patients with type 2 diabetes vs. 80.3% in patients without diabetes, P < 0.05).

In our study, patients with diabetes who underwent a CABG had higher CCI values than those without diabetes (40.8% vs. 36.3% with one or more comorbidities).

In patients with type 2 diabetes, the IHM was significantly lower than in those without diabetes (5.8% vs. 7.9%); however the median LOS was significantly higher in patients with type 2 diabetes (16 days [IQR 16 days] compared with those without diabetes (15 days [IQR 15 days]).

**Figure 3:**
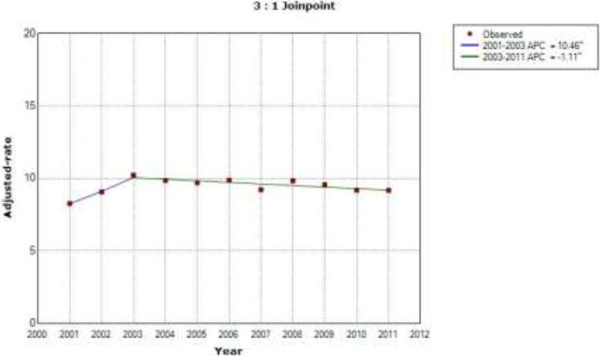
**Joinpoint analysis in annual CABG in patients with type 2 diabetes in Spain, 2001–2011.** Footnote: APC: Annual percent change (based on rates that were sex and aged-adjusted using the Spanish National Statistics Institute Census projections) calculated by using joinpoint regression analysis. ˆAPC is significantly different from zero (two-side P < 0.05).

**Figure 4:**
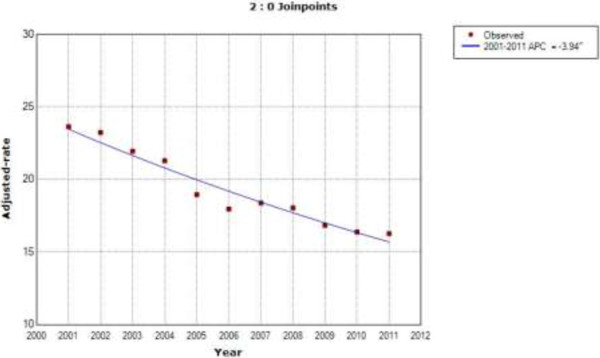
**Joinpoint analysis in annual CABG in patients without type 2 diabetes in Spain, 2001–2011.** Footnote: APC: Annual percent change (based on rates that were sex and aged-adjusted using the Spanish National Statistics Institute Census projections) calculated by using joinpoint regression analysis. ˆAPC is significantly different from zero (two-side P < 0.05).

Among patients with type 2 diabetes who underwent CABG, the sex and age-adjusted CABG incidence rate increased by 10.4% per year from 2001 to 2003, then decreased by 1.1% through 2011 (Figure 3). In patients without diabetes, the incidence rate decreased at a constant rate of 3.94% per year over the entire period of study (Figure 4).

As can be seen in Table 3, over the 11-year study period, the mean age of patients with type 2 diabetes who underwent CABG was 66.1 ± 8.6 years in 2001 and 67.8 ± 8.7 years in 2011. Significant differences in age were observed (P < 0.01). In 2001 the proportion of men was 70.8%, which rose to 76.4% in 2011 (P < 0.05).

**Table 3:** Characteristics and outcomes of hospital discharges after coronary artery bypass graft among patients with and without type 2 diabetes in Spain, 2001–2011

		**2001**	**2002**	**2003**	**2004**	**2005**	**2006**	**2007**	**2008**	**2009**	**2010**	**2011**
No diabetes	N	5487	5445	5258	5203	4728	4558	4753	4746	4492	4416	4434
	Incidence*	23.6	23.2	21.9	21.2	18.9	17.9	18.3	18.0	16.8	16.3	16.2
	Age§, mean	65.4	65.2	65.6	66.1	66.6	66.9	66.8	66.9	67.0	67	67.6
	(SD)	(9.8)	(9.8)	(9.9)	(9.9)	(9.8)	(10.0)	(10.0)	(10.1)	(10.2)	(10.4)	(10.3)
	Female ǂ, n	1055	1038	998	1043	949	869	958	978	872	921	865
	(%)	(19.2)	(19.0)	(18.9)	(20.0)	(20.0)	(19.0)	(20.1)	(20.6)	(19.4)	(20.8)	(19.5)
	CCI 0ǂ, n	3889	3813	3536	3405	2983	2830	2909	2857	2607	2626	2605
	(%)	(70.8)	(70.0)	(67.2)	(65.4)	(63.0)	(62.0)	(61.2)	(60.2)	(58.0)	(59.4)	(58.7)
	CCI 1-2ǂ, n	1552	1572	1649	1714	1656	1642	1750	1801	1784	1670	1713
	(%)	(28.2)	(28.8)	(31.3)	(32.9)	(35.0)	(36.0)	(36.8)	(37.9)	(39.7)	(37.8)	(38.6)
	CCI ≥ 3ǂ, n	46	60	73	84	89	86	94	88	101	120	116
	(%)	(0.8)	(1.1)	(1.3)	(1.6)	(1.8)	(1.8)	(1.9)	(1.8)	(2.2)	(2.7)	(2.6)
	LOS§, median	16.0	16.0	16.0	15.0	16.0	16.0	16.0	15.0	15.0	14.0	15.0
	(IQR)	(16.0)	(15.0)	(14.0)	(15.0)	(16.0)	(13.0)	(15.0)	(14.0)	(14.0)	(12.0)	(14.0)
	IHM ǂ, n	526	463	442	395	361	358	374	392	312	314	300
	(%)	(9.5)	(8.5)	(8.4)	(7.5)	(7.6)	(7.8)	(7.8)	(8.2)	(6.9)	(7.1)	(6.7)
Type 2 diabetes	N	1937	2159	2473	2425	2429	2527	2393	2587	2555	2479	2502
	Incidence*	8.2	9.0	10.2	9.8	9.6	9.8	9.2	9.8	9.5	9.1	9.1
	Age§, mean	66.1	66.8	66.7	67.0	67.2	67.7	67.3	67.4	67.5	68.0	67.8
	(SD)	(8.6)	(8.4)	(8.4)	(8.7)	(8.3)	(8.6)	(8.8)	(8.8)	(8.8)	(8.9)	(8.7)
	Female ǂ, n	566	634	743	681	695	663	598	639	640	636	589
	(%)	(29.2)	(29.3)	(30.0)	(28.0)	(28.6)	(26.2)	(24.9)	(24.7)	(25.0)	(25.6)	(23.5)
	CCI 0ǂ, n	1315	1395	1549	1484	1448	1533	1371	1454	1398	1343	1362
	(%)	(67.8)	(64.6)	(62.6)	(61.2)	(59.6)	(60.6)	(57.2)	(56.2)	(54.7)	(54.1)	(54.4)
	CCI 1-2ǂ, n	600	730	885	893	937	952	967	1074	1096	1064	1070
	(%)	(30.9)	(33.8)	(35.7)	(36.8)	(38.5)	(37.6)	(40.4)	(41.5)	(42.9)	(42.9)	(42.7)
	CCI ≥ 3ǂ, n	22	34	39	48	44	42	55	59	61	72	70
	(%)	(1.1)	(1.5)	(1.5)	(1.9)	(1.8)	(1.6)	(2.3)	(2.2)	(2.3)	(2.9)	(2.8)
	LOS§, median	18.0	18.0	17.0	16.0	16.0	17.0	16.0	16.0	15.0	15.0	14.0
	(IQR)	(17.0)	(17.0)	(16.0)	(16.0)	(16.0)	(15.0)	(16.0)	(15.0)	(14.0)	(14.0)	(14.0)
	IHM ǂ, n	138	172	144	149	168	160	132	135	112	118	112
	(%)	(7.1)	(7.9)	(5.8)	(6.1)	(6.9)	(6.3)	(5.5)	(5.2)	(4.3)	(4.7)	(4.4)

N: Number of procedures; LOS: length of stay; IHM: In-hospital mortality; CCI (Charlson Comorbidity Index): Comorbidities included in the Charlson comorbidity index, except diabetes and AMI.

Incidence per 100.000. Incidence was calculated using the Spanish National Statistics Institute census projections adjusted by sex and age (16).

*P < 0.05 (Poisson regression analysis).

§P < 0.05 (ANOVA or Kruskal-Wallis analysis).

ǂP < 0.05(χ2 linear tend analysis).

In our study, LOS in patients with type 2 diabetes decreased significantly from 18 days (IQR, 17 days) in 2001 to 14 days (IQR, 14 days) in 2011. In patients without diabetes, LOS also decreased significantly over the period of study (Table 3).

The IHM decreased significantly for those with and without diabetes during the 11-year study period (7.1% in 2001 vs. 4.4% in 2011 and 9.5% vs. 6.7%, respectively) (Table 3).

After multivariate adjustment, the IHM among patients with type 2 diabetes who underwent a CABG decreased significantly over the entire study period (OR 0.93; 95% CI 0.91-0.94), was significantly higher in women than in men (OR 1.69; 95% CI 1.52-1.89) and in those with one or two (OR 2.58; 95% CI 2.31-2.88) and with three or more (OR 4.95; 95% CI 3.83-6.42) comorbidities. Patients suffering from type 2 diabetes who underwent a CABG had a 0.67 (95% CI 0.63-0.71) times lower probability of dying during hospitalization than those without diabetes (Table 2).

## Discussion

Using the Spanish National Hospital Database, we found different trends over the last 11 years in the hospitalizations of subjects with and without type 2 diabetes who underwent coronary revascularization procedures.

Our results reveal that patients with type 2 diabetes account for 29.8% of all revascularization procedures in Spain. We found an increase in PCI procedure rates from 2001 to 2011; and a decline in hospital admissions for CABG in patients with type 2 diabetes from 2003 to 2011.

Our national results are consistent with other studies indicating that PCI rates have increased significantly due to advances in stent device technology and adjunctive pharmacology and CABG rates have declined due to the fact that this procedure is more invasive than PCI procedures [14,21,22]. Rana et al. indicated that drug-eluting stents were used more often in patients with severe comorbidities and multivessel disease [23], but a recent study indicated that CABG in patients with diabetes and coronary artery disease offers advantages in terms of survival [24]. Another advantage of CABG procedures is with regard to the need for repeat revascularization. Contini et al. concluded that only 51.3% of PCI diabetic patients underwent “complete” revascularization, while 85.6% of CABG patients with type 2 diabetes did so [25].

We found that IHM increased over time among non-diabetic patients after PCI. Vamos et al. observed that, among those without diabetes from 2004–5 to 2009–10, the percentage of patients who underwent a PCI and died in the hospital increased from 0.9% to 1.5% (p < 0.001). These authors suggest that this increase, despite technological advances in interventional techniques and improvements in periprocedural care, may be attributable to the increasing complexity of cases referred for PCI [14]. We agree with this interpretation, since in our population the CCI was significantly worse in 2001 than in 2011 (CCI ≥1; 22.5% and 29.7% respectively). Another possible explanation is that over the study period, in the group of patients without diabetes, there may have been an increase in the prevalence of subjects with undiagnosed glucose abnormalities, and consequently,with a higher risk of adverse cardiac effects [26,27]. Kassain et al., 2012 concluded that the risk of major adverse cardiovascular events following a PCI in diabetics with good glycemic control (HBA1c ≤ 7%) was not significantly different from that of non-diabetics (adjusted HR = 1.33;95% CI:0.38 to 4.68, P = 0.66) [28].

In our study, the IHM remained stable over time among diabetic patients with a PCI. The higher comorbidity and older age can partially explain this lack of improvement.

Holper et al. found significant improvements in mortality rates over time (9.7% in 1997–1998; 6.5% in 1999; 4.1% in 2001–2002; 5.4% in 2004 and 4.7% in 2006) in patients with diabetes treated with oral agents after a PCI [29].

On the other hand, Vamos et al. found significant increases in IHM rates for PCI from 2004 to 2010 [14].

A recent study in United States concluded that the mortality at 3-year follow-up after PCI, of patients with diabetes treated with oral agents, had significantly higher adjusted hazards of death (HR: 1.32 [95% CI: 1.29 to1.35] compared to nondiabetic patients. The authors indicated that the mechanisms for this incremental risk are likely multifactorial: a greater underlying burden of atherosclerosis, microvascular disease, a prothrombotic state, more neointimal hyperplasia, greater vascular inflammation, and/or further accumulation of diabetes-related end-organ damage and comorbidities during the follow-up period [30].

Incomplete revascularization is frequently the final outcome in patients with multivessel coronary disease who undergo PCI [25]. However, we found a significant decline in IHM in CABG patients. Hassan et al. (2010) explain that the greater improvement in CABG in-hospital mortality outcome versus PCI may be attributable to advanced myocardial protection techniques, superior perioperative critical care, and improved patient selection and surgical timing than in PCI patients [31].

Among those who underwent a CABG, we found that IHM was higher in patients without diabetes than in those with type 2 diabetes. In England, the IHM found in years 2009–10 among non-diabetic patients who underwent a CABG was 3.1% compared to 2.8% among diabetic patients [14]. However the reasons for these differences require further investigations with clinical data and longer follow-up outside the hospital.

In Canada, Elbarouni B et al. found that diabetic patients continue to experience worse outcomes, including IHM, when compared to those without diabetes. Paradoxically, these diabetic patients are treated with less invasive treatments compared to their nondiabetic counterparts [32]. Also in the same country, Kang et al. found that, among subjects who had antianginal use prior to non-ST-elevation acute coronary syndrome, the prevalence of diabetes was 36.5% and only 20.7% among those not using these medications. Furthermore, patients on chronic antianginal therapy before admission had lower rates of catheterization during hospitalization. This latter observation may be related to the fact that this group had significantly more comorbidities, as well as a higher burden of coronary disease at baseline than had been previously delineated, and might not be suited to revascularization [33].

In our investigation, the median LOS for PCI decreased from 7 days in 2001 to 6 days in 2011, among diabetic patients, and from 6 to 5 days among non-diabetics. With regard to CABG, the median was 18 days in 2001 and 14 days in 2011 for those suffering from diabetes, with equivalent figures of 16 and 15 days for those without the disease. It is remarkable that these figures are almost twice those reported by Vamos et al., in England, who found a median of 2 days for PCI and 9 days for CABG among those with and without diabetes in the 2009–10 period [14].

In Spain and in England, the medical insurance systems are very similar in terms of universal coverage, being funded by taxes and predominantly operating within the public sector [34,35]. Thus, we think these large differences may be explained by several reasons. First, the differences in the percentage of scheduled/emergency procedures between the two countries; with a much higher percentage of scheduled procedures in England. Second, the LOS in our investigation was calculated from the time the patient was admitted to the hospital and not from the time that the procedure was performed. We do not know whether the data for England was analyzed in the same way or whether they calculated only from the moment the procedure was performed until the patient was discharged. Finally, the severity of the CAD or the comorbidities was different between the two countries.

In line with the results discussed for LOS, we also find that IHM after CABG in Spain was higher than in England. With figures of 4.7% and 7.1% among diabetic and non-diabetic patients in the year 2010 in Spain, and corresponding figures for England of 2.8% and 3.1% in the 2009–10 period. These differences cannot be explained by patient characteristics, which are similar with regard to age and sex. Beside the aforementioned, another possible explanation for the improvement in England is that the prevention and treatment of cardiovascular disease and its risk factors have assumed increasing importance in UK health policy over the last decade; investments have been made in health services; the introduction of a wide range of initiatives; national treatment standards for the management of major chronic conditions with a special focus on secondary prevention [14,22].

In type 2 diabetes patients who had undergone either a PCI or a CABG, women had worse outcomes than men. Our results are consistent with those of previous studies, which suggest that the worse effect of diabetes on outcomes in women might be related to the onset mechanism for cardiovascular disease, the success of the revascularization coronary procedure, and the higher burden of cardiovascular risk factors [21,36-38]. However, the worse results from these procedures among women call for urgent investigations to identify and reduce these significant differences.

The strength of our investigation lies in its large sample size, its 11-year follow-up period and its standardized methodology, which has previously been used to investigate diabetes and its complications in Spain and elsewhere [39,40]. Nevertheless, our study is subject to a series of limitations. Our data source was the CMBD, an administrative database that contains discharge data for Spanish hospitalizations and uses information the physician has included in the discharge report; therefore, it does not include all the variables of the clinical history such as the severity of the coronary disease or other chronic conditions, the duration of diabetes complications or treatment. Another limitation of this database is its anonymity (no identifying items such as clinical history number), which makes it impossible to detect whether the same patient was admitted more than once during the same year. In addition, patients who moved from one hospital to another would appear twice.

Nevertheless, this dataset, which was introduced in Spain in 1982, is a mandatory register, and its coverage is estimated to be greater than 95% [15]. We were unable to calculate diabetes-specific incidence rates, because no studies in Spain cover blood glucose measurements or HbA1C for the entire population; consequently, no precise estimate of the prevalence of diabetes is available [41]. Concerns have been raised about the accuracy of routinely-collected datasets; however, these datasets are periodically audited. Consequently, the quality and validity of our dataset has been assessed and shown to be useful for health research [42].

Another limitation of our study is the missing causal relationship between IHM and CAGD/PCI as cancer and non-cardiovascular admitted patients were also included, the mortality of whom may not have been affected by the CABG/PCI.

## Conclusions

Our results show that the annual percentage change in PCI procedures increased in diabetic and non-diabetic patients. Outcomes such as LOS is worse among individuals with diabetes than those without diabetes for PCI although they improved over the entire study period for both groups. Higher comorbidity and female gender are associated with higher IHM in PCI procedures.

We found a decrease in the use of CABG procedures in patients with and without type 2 diabetes. IHM was higher in patients without type 2 diabetes than those with it.

Given the rapid increase in the prevalence of diabetes and the aging population, these findings emphasize the need for further improvement in the control of cardiovascular risk factors in people with diabetes.

## Abbreviations

AMI: Acute myocardial infarction; APC: Annual percentage of change; CABG: Coronary artery bypass graft surgery; CAD: Coronary artery disease; CCI: Charlson comorbidity index; CMBD: Spanish Minimum Basic Data Set (*Conjunto Mínimo Básico de Datos)*; ICD-9-CM: International Classification Diseases-Ninth Revision, Clinical Modification; IHM: In-hospital mortality; LOS: Length of stay; PCI: Percutaneous coronary intervention.

## Competing interests

The authors declare that they have no competing interests.

## Authors’ contributions

AL and PCG researched data, contributed to the discussion, wrote the manuscript, and reviewed/edited the manuscript. RJG contributed to the discussion, wrote the manuscript, and reviewed/edited the manuscript. VHB researched data and reviewed/edited the manuscript. NPF, JMMY, MMB, IJT, AGM and CGP contributed to the discussion and reviewed/edited the manuscript. All authors reviewed and gave their final approval of the version to be submitted.
